# Impact of the COVID-19 pandemic on levels of deep-ocean acoustic noise

**DOI:** 10.1038/s41598-023-31376-3

**Published:** 2023-03-21

**Authors:** Stephen Robinson, Peter Harris, Sei-Him Cheong, Lian Wang, Valerie Livina, Georgios Haralabus, Mario Zampolli, Peter Nielsen

**Affiliations:** 1 National Physical Laboratory, Hampton Road, Teddington, TW11 0LW UK; 2 Hydro-Acoustics, Engineering and Development Section, IMS, CTBTO, Vienna, Austria; 3 Esbjerg, Denmark

**Keywords:** Ocean sciences, Marine biology, Physical oceanography

## Abstract

The extraordinary circumstances of the COVID-19 pandemic led to measures to mitigate the spread of the disease, with lockdowns and mobility restrictions at national and international levels. These measures led to sudden and sometimes dramatic reductions in human activity, including significant reductions in ship traffic in the maritime sector. We report on a reduction of deep-ocean acoustic noise in three ocean basins in 2020, based on data acquired by hydroacoustic stations in the International Monitoring System of the Comprehensive Nuclear-Test-Ban Treaty. The noise levels measured in 2020 are compared with predicted levels obtained from modelling data from previous years using Gaussian Process regression. Comparison of the predictions with measured data for 2020 shows reductions of between 1 and 3 dB in the frequency range from 10 to 100 Hz for all but one of the stations.

## Introduction

The COVID-19 pandemic, discovered in late 2019 and announced as a global pandemic by the World Health Organization (WHO) in March 2020^1^, led to widespread measures to mitigate the spread of the disease. These measures led to sudden and sometimes dramatic reductions in human activity in sectors such as transport, industry, energy, tourism, and construction. A number of studies have reported on the resulting changes in the scale and extent of human impacts on the natural environment^2,3^, covering fields such as seismology^4,5^, air pollution^6^, carbon dioxide concentrations^7^, and urban noise pollution^8,9^.

The mitigating measures had a widespread effect on human activity in the oceans, with radical changes reported in marine shipping traffic for tourism, fisheries, cargo, energy and transportation, the latter alone accounting for more than 80% of world trade. Studies of the changes in traffic have been based on data from Automated Information Service (AIS) transponders on vessels in the global ocean and on statistics of port calls by vessels^10–12^. Some of the greatest reductions in traffic were observed in the spring and summer months of 2020 (from March to June), when severe restrictions were in place globally, with (for example) up to 13% reduction in container ships and up to 42% reduction in passenger ships^11^. Vessel traffic reductions were reported in nearly 44% of the global oceans and in 77.5% of national waters during April 2020^10^.

As a consequence of the reduction in traffic, the environmental pressures and stressors associated with shipping, such as greenhouse gas emissions and underwater noise, were widely expected to decrease^13^. Anthropogenic underwater noise has long been identified as a pressure on the marine environment^14^, with shipping noise providing a strong contribution to the ambient soundscape at frequencies below 500 Hz^15–17^ where, under the right conditions, sound suffers little propagation loss due to a low absorption and can thus propagate over considerable distances^18^. Sound which couples into the deep sound fixing and ranging (SOFAR) channel can propagate even farther due to the reduced interaction with the surface and the seafloor^18,19^.

The potential for reduction in ocean ambient noise during the COVID-19 pandemic has led to a number of recent studies based around the north American coast: the sound pressure levels close to the Port of Vancouver have been shown to have decreased by 1.5 dB during early 2020^20^; measurements off the Oregon coast of the USA showed the sound pressure level in the 63 Hz one-third-octave band reduced in the spring of 2020 by about 1.6 dB compared to the previous five years^21^; reductions were observed in underwater noise and vessel traffic in the approaches to Halifax Harbor, Canada^22^; and reduction of low-frequency vessel noise was observed in Monterey Bay national marine sanctuary during the pandemic^23^. Recent studies looking at the North Sea showed a decline in sound pressure of 13% (reduction in sound pressure level of 1.2 dB) for frequencies in the range 10 Hz to 1 kHz corresponding to reduced level of shipping activity^24^. Some studies focused on local changes in soundscape have ascribed the reductions in sound levels to a pandemic-induced reduction in vessel speed rather than vessel numbers^25^, and significant local reductions have been observed where transport links such as ferries shut down during the pandemic^26^.

**Figure 1:**
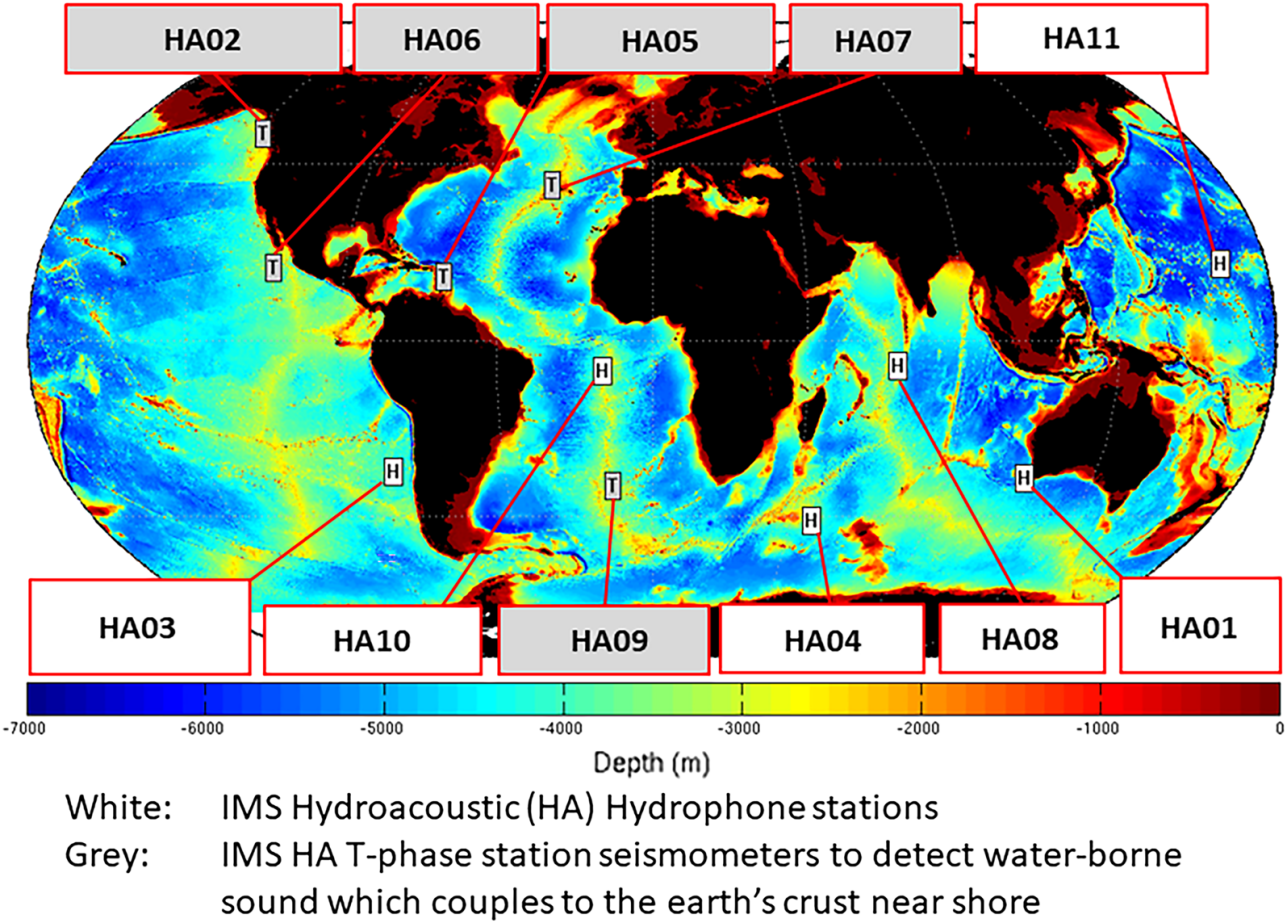
Locations around the globe of the eleven hydroacoustic stations of the CTBT IMS comprising six hydrophone stations (labelled in white) and five seismic T-phase stations (labelled in grey). The hydrophone stations considered in this study are Wake Island (HA11) located in the Western Pacific Ocean, Ascension Island (HA10) located in the South Atlantic Ocean, Diego Garcia Island located in the Indian Ocean (HA08), Juan Fernandez Islands located in the South Pacific Ocean (H03N), and Cape Leeuwin (H01W) located off the southwest shore of Western Australia. (Courtesy of CTBTO—reproduced by permission).

The above studies examine the changes in sound levels in coastal areas and enclosed shallow seas, but as yet there has been no report of observations in the deep ocean during the pandemic. The objective of the study reported here is to examine the changes in low frequency sound levels in the deep ocean by use of the data acquired by hydroacoustic stations in the International Monitoring System (IMS) of the Comprehensive Nuclear-Test-Ban Treaty (CTBT). The complete IMS consists of a global network of 337 stations and laboratories that use four complimentary verification technologies: seismic, infrasound, radionuclide and hydroacoustic. As of the time of writing of this paper the IMS is 90% complete. The hydroacoustic component of the IMS comprises 11 hydroacoustic stations (6 hydrophone and 5 seismic T-phase stations) located around the globe^27–29^: see Fig. 1. The IMS hydroacoustic network is the first component of the IMS to be fully certified as of 2017.

There have been numerous studies looking at deep ocean noise and its long-term trends. Many of the examples, ranging from several decades ago to the early twentieth century, centred around the North Pacific Ocean^30–33^. However, a number of recent studies have made use of the data from the more widely distributed CTBT IMS monitoring stations^34–38^. Many of these studies have shown gradual long-term variations in low-frequency sound levels^34,38^, but also annual variations in the levels associated with seasonal effects^33,38^.

In the study described here, we report an analysis of the sound levels in the deep ocean from data recorded by the CTBT IMS hydroacoustic stations during 2020 and in the preceding years to determine whether a reduction in levels is observable. One method to evaluate any potential changes in 2020 during the COVID-19 pandemic might be to examine the data for the same period in successive years (the same days or weeks in successive years) and look for changes. However, there are difficulties in applying this method because there are confounding factors which can also influence the year-to-year variations. These include (1) gradual long-term trends in the data evident from a number of other studies^34,38^; (2) seasonal fluctuations in the data due to changes in the sources or environment over an annual cycle, again evident from previous studies^33,38^; (3) short term fluctuation in the data due to events lasting minutes, hours or days, such as for example earthquake aftershock sequences and undersea volcanism.

The method chosen for this study is to represent the data for the period up to the end of 2019 using a model which accounts for the factors listed above, then use the model to predict sound levels in 2020 and compare those predictions with the actual measured data. If the differences between the predicted and measured values are large compared to the uncertainty in those differences, then there is evidence that sound levels in 2020 have changed compared to previous years. On the other hand, if the predicted and measured values are consistent, accounting for uncertainty, then there is no evidence for a change. It is difficult to use parametric physics-based models because the processes that generate the data are too complicated to be described in this way. Furthermore, the long-term and seasonal features in the data do not remain constant but evolve with time, making it difficult to use parametric empirical models such as straight lines, polynomial functions, and Fourier series^38,39^. This motivated the choice of Gaussian Process regression for the modelling, a machine learning method which provides a flexible way of modelling data that is not limited by a fixed functional form, and which also has the benefit of providing uncertainties in predictions made using the model^40,41^.

This paper presents for the first time a comprehensive analysis of the deep-ocean acoustic noise levels during 2020 and the preceding years, demonstrating a significant reduction in noise levels for the first half of 2020 for all but one of the CTBT IMS hydroacoustic stations considered, with the levels being largely restored by the end of 2020. The use of the data from five of the six CTBT IMS hydroacoustic stations has enabled the analysis to be applied to three ocean basins (Pacific, Atlantic and Indian), integrating the low-frequency sound radiated from distant sources such as remote shipping lanes. The exception was the station at Cape Leeuwin which showed no significant reduction. The use of Gaussian Process regression has provided a flexible way of modelling data to account for evolving seasonal and long-term trends, providing uncertainties in the observed reductions. The observed reductions of between 1 and 3 dB in the frequency range from 10 to 100 Hz correlate strongly with independent reports of reductions in ship traffic suggesting that the downturn in maritime activity may be partly a cause, though the contributions to the changes from other sound sources cannot be ruled out. Further investigation of the correlation between ship traffic in the vicinity of the IMS stations is planned as well as the influence of changes in geophysical surveying activity.

## Results

### Data reduction

Each CTBT IMS hydroacoustic station consists of two pairs of three hydrophones, i.e., “triplets”, placed approximately 2 km apart at depths near the axis of the SOFAR channel, close to where the vertical sound speed profile exhibits a minimum, on either side of an island (HA01 in Australia is the only station with one triplet). The locations of the stations allow good spatial coverage of the world’s oceans by taking advantage of the low absorption of low-frequencies, and the long-range SOFAR channel sound propagation in the deep ocean. The sampling frequency for the sound pressure recordings is 250 Hz to provide information at acoustic frequencies up to 100 Hz, and a bit depth of 24 bits yields a maximum theoretical dynamic range of approximately 144 dB (the achievable dynamic range being limited by the self-noise of the system, with a minimum of 120 dB the operational requirement).

The data analysed in this study come from hydrophones of the north triplet of Wake Island (H11N) located in the Western Pacific Ocean, the north triplet of Ascension Island (H10N) located in the South Atlantic Ocean, the south triplet of Diego Garcia Island located in the Indian Ocean (H08S), the north triplet of Juan Fernandez Islands located in the South Pacific Ocean (H03N), and the only triplet at Cape Leeuwin (H01W) located off the southwest shore of Western Australia. The locations around the globe of the hydroacoustic stations are shown in Fig. 1 and information about the selected hydrophones and the data collected by them is presented in Table 1. The hydrophones in the triplets at Crozet Islands (H04N and H04S) in the South Indian Ocean are not considered because of their relatively short record length compared to the other stations.

**Table 1:** Information about the hydrophones H01W1 at the CTBT station at Cape Leeuwin, H03N1 at Juan Fernandez Islands, H08S1 at Diego Garcia Island, H10N1 at Ascension Island, and H11N1 at Wake Island.

Code	Latitude/°N	Longitude/°E	Depth/km	Water depth/km	Starting date for time series	Number of points in time series (number of weeks)
H01W1	− 34.892 99	114.153 98	1.042	1.535	02-Jan-2003	928
H03N1	− 33.458 02	− 78.934 14	0.812	1.537	22-Apr-2014	349
H08S1	− 7.645 30	72.474 40	1.413	1.890	02-Jan-2003	875
H10N1	− 7.845 673	− 14.480 232	0.842	1.925	28-Mar-2005	795
H11N1	19.713 56	166.891 09	0.731	1.418	16-Dec-2007	679

The amount of data collected by each hydrophone over its operating period is of the order of terabytes. The process of reducing the data to a time series for analysis is described elsewhere^38^ and only summarised here. The process starts by scaling the raw data in the form of A/D counts to values of sound pressure using the hydrophone sensitivity at 10 Hz and filtering the scaled data in the frequency domain using the inverse frequency response of the hydrophone channel in order to eliminate artefacts such as low-frequency roll-off introduced by the recording instrumentation. The filtered data are then collected into 1-min intervals and the data in each interval are filtered into the following frequency bands: 10–100 Hz (broadband), 1–10 Hz (very low), 10–40 Hz (low), 40–70 Hz (medium) and 70–100 Hz (high). The mean-squared sound pressure within each frequency band is calculated for each 1-min interval and the resulting values are divided by the bandwidth to be expressed as values of sound pressure spectral density level (SPSDL)^42^ in units of dB rel μPa^2^/Hz. The low, medium, and high frequency bands are chosen somewhat arbitrarily to break up the frequency range and are intended as being very broadly descriptive of the sounds from different groups of sources, both anthropogenic and natural.

The 1-min values of SPSDL are then aggregated over selected time periods, such as days, weeks or months, and statistical percentile levels are calculated from the distributions of the values of SPDSL over those time periods. The focus in this study is on the 10th percentile, denoted by $P_{10}$, of the distributions as it represents low levels of acoustic noise and may be considered as representative of “background” sound levels generated by distant sources such as remote shipping. In contrast, higher percentiles, such as $P_{90}$ and $P_{99}$, are strongly affected by high amplitude short-term events often from sources close to the hydrophones, such as biological sources. For the results shown in this paper, the selected percentiles are calculated for a weekly aggregation interval that is chosen to reduce the sheer volume of the data and provide some smoothing (compared to daily data) without losing trend-related information.

Finally, the last step of the data reduction process is to remove outliers associated with anomalously high values of SPSDL that are identified as being non-acoustic, for example, corresponding to electrical calibration signals transmitted as part of periodic system checks. The data sequences available for analysis can have some short gaps in the data where outliers have been removed or where occasional signal dropouts have occurred, and this can generate challenges for some methods of time series analysis. Unlike these methods, the data analysis technique used in this work does not require time series that are complete and free of missing values.

### Detailed results for hydrophone H11N1 at Wake Island

Let $y\left( t \right)$ denote SPSDL at time $t$ for a given hydrophone, frequency band (e.g., 10–40 Hz, 40–70 Hz, or 70–100 Hz), aggregation period (e.g., week) and statistical level (e.g., $P_{10}$). Furthermore, let $\left( {t_{i} ,y_{i} } \right),i = 1, \ldots ,m,$ where $y_{i}$ is an observed value of $y\left( {t_{i} } \right)$, denote data collected up until the end of 2019, and $\left( {\tau_{j} ,\psi_{j} } \right),j = 1, \ldots ,n,$ denote data collected during 2020. The data collected up until the end of 2019 is used to obtain predicted values $\hat{y}_{j}$ of SPSDL at times $\tau_{j}$ during 2020 and those predicted values are then compared with the observed values $\psi_{j}$. To enable a decision to be made about the statistical significance of the differences between observed and predicted values it is necessary to account for uncertainties in those values.

Gaussian Process (GP) regression applied to the data $\left( {t_{i} ,y_{i} } \right),i = 1, \ldots ,m,$ collected up until the end of 2019 is used to provide the predicted values and the uncertainties in those values. The GP model for the regression comprises three components representing characteristics of SPSDL at, respectively, long, medium (seasonal), and short timescales. The GP is not intended to represent a physical model of the mechanisms and processes that generate a time series of SPSDL values, rather it provides an empirical description of the different characteristics observed in such a time series. So, the long-term component is used to represent gradual long-term changes at timescales longer than one year, the medium-term component to represent periodic, seasonal fluctuations due to changes in sound sources or the environment over one year, and the short-term component to represent fluctuations due to events lasting over time periods less than one year, such as earthquake aftershock sequences and undersea volcanism. Finally, terms representing uncorrelated random effects are used to capture any remaining discrepancies between values of the GP model and the observed SPSDL values.

To illustrate the GP modelling process, detailed results are first presented for hydrophone number 1, named H11N1, in the northern triplet of the CTBT Wake Island station and using data for the frequency band 10–40 Hz, a weekly aggregation period and the statistical level $P_{10}$. Final results are then shown for the other frequency bands for that hydrophone and all frequency bands for a hydrophone at each of the other stations (except the station HA04 at Crozet Islands).

**Figure 2:**
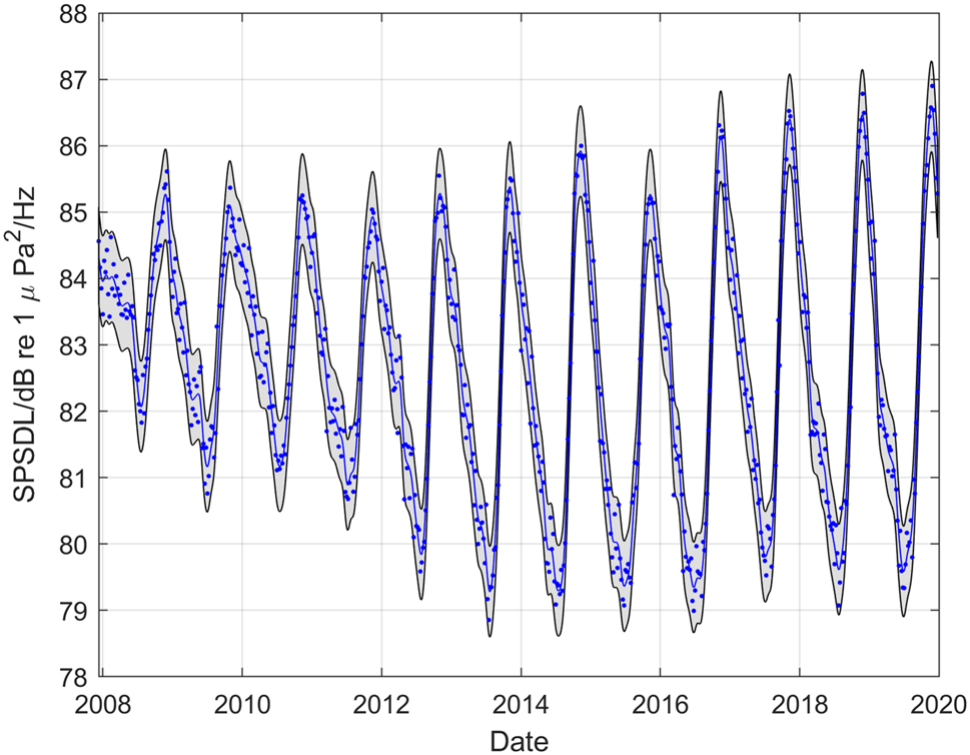
GP regression for the northern triplet hydrophone H11N1 data recorded at the CTBT Wake Island station for the frequency band 10–40 Hz, a weekly aggregation period and the statistical level *P*_10_.

**Figure 3:**
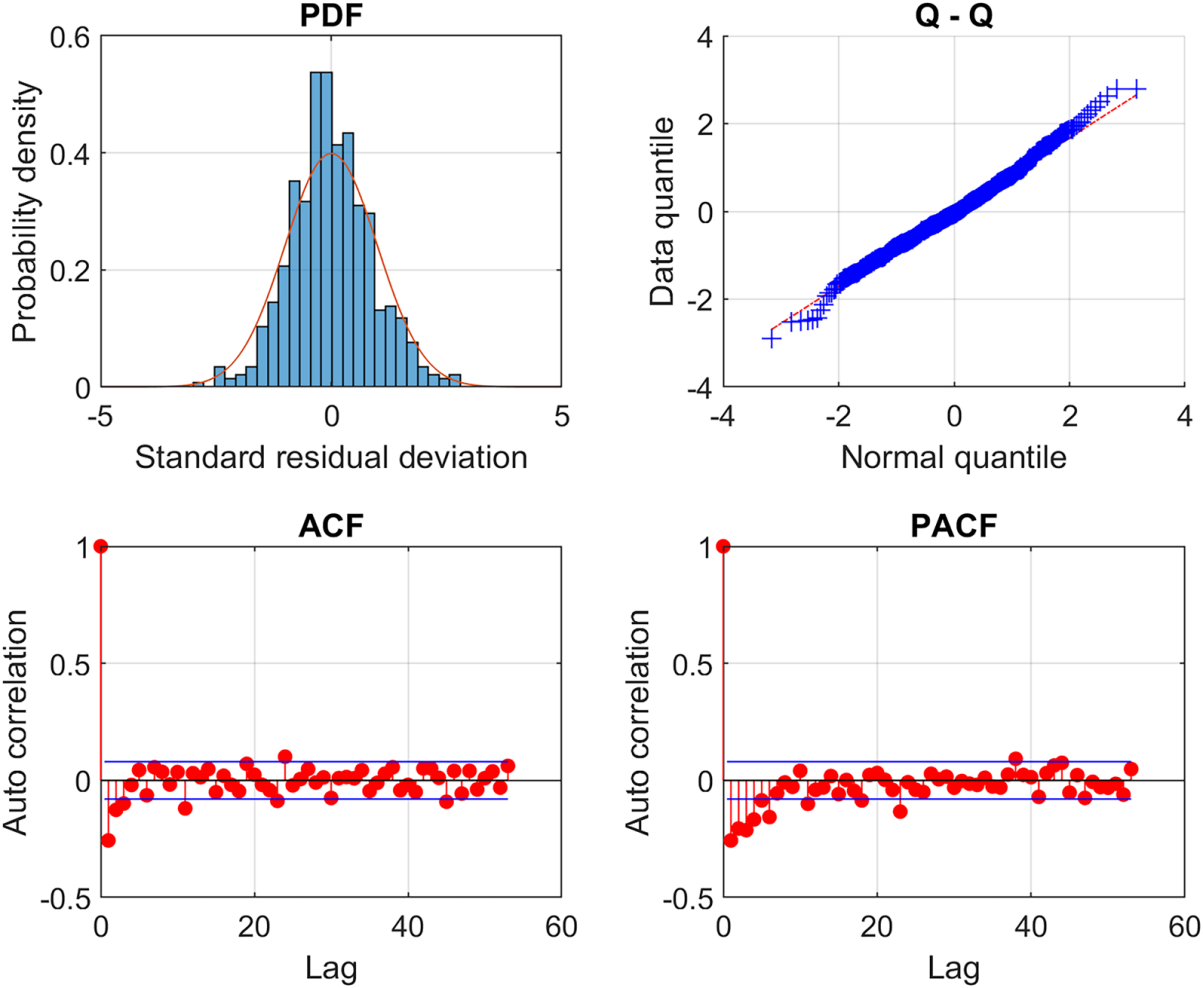
Diagnostics for the standardised residual deviations corresponding to the results of the GP regression shown in Fig. 2. A scaled histogram of those deviations compared against the probability density function for the standard normal distribution N(0, 1), a QQ-plot of the quantiles of the deviations compared against the quantiles of N(0, 1), and the sample autocorrelation (ACF) and sample partial autocorrelation (PACF) functions for various lags.

Figure 2 shows (as dots) the observed weekly SPSDL data in the period January 2008 to December 2019 during which the hydrophone was operational, together with predicted values $\hat{y}\left( t \right)$ for SPSDL at daily intervals during that period (joined to form a curve) and an uncertainty band with lower and upper curves defined by values $\hat{y}\left( t \right) \pm 2u\left( t \right)$, where $u\left( t \right)$ is the standard uncertainty of $\hat{y}\left( t \right)$. The predicted values $\hat{y}\left( t \right)$ and the standard uncertainties $u\left( t \right)$ are provided by the GP model fitted to the data $\left( {t_{i} ,y_{i} } \right),i = 1, \ldots ,m,$ collected up until the end of 2019. Figure 3 presents various diagnostics for the standardised residual deviations defined by

$$r_{i} = \frac{{y_{i} - \hat{y}(t_{i} )}}{{\hat{\sigma }_{0} }}, i = 1, \ldots ,m,$$

where $\hat{\sigma }_{0}$ is an estimate of the standard deviation of uncorrelated random effects influencing the observed data. These diagnostics indicate that the distribution of the deviations is comparable to the form of a zero-mean Gaussian that is assumed to describe the uncorrelated random effects, and the autocorrelations of the deviations are small for different time lags, which together provide evidence to accept the adequacy of the fitted GP model.

**Figure 4:**
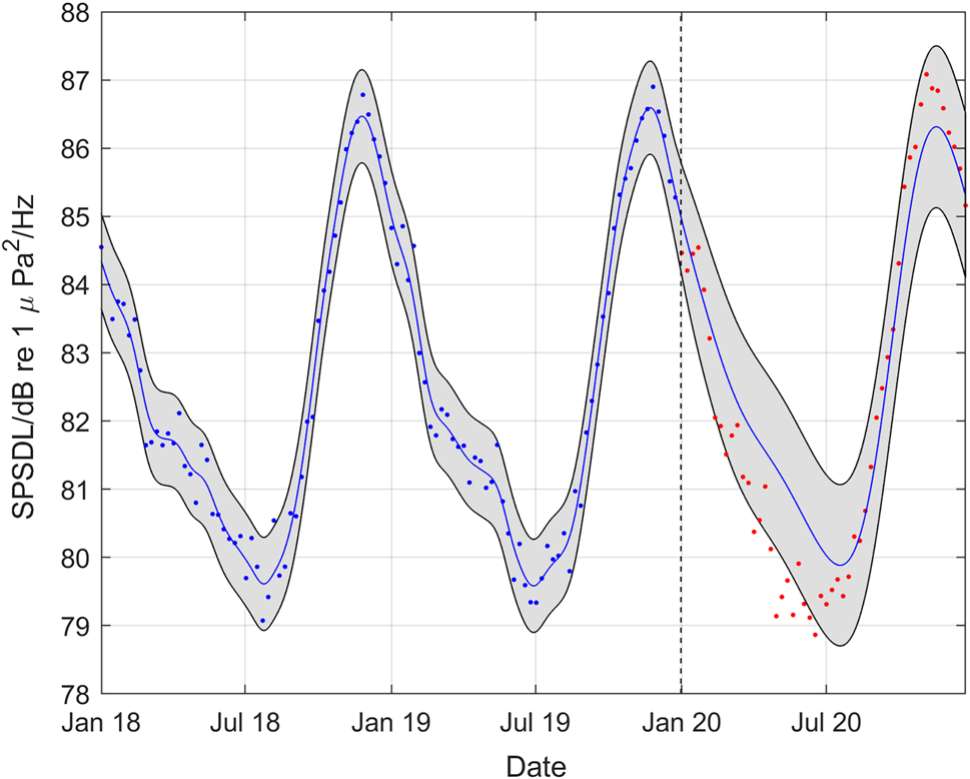
Part of Fig. 2 restricted to the period January 2018 to December 2019, showing also the observed weekly SPSDL data in 2020 and the predictions of SPSDL in 2020 provided by the GP with their corresponding uncertainty band.

**Figure 5:**
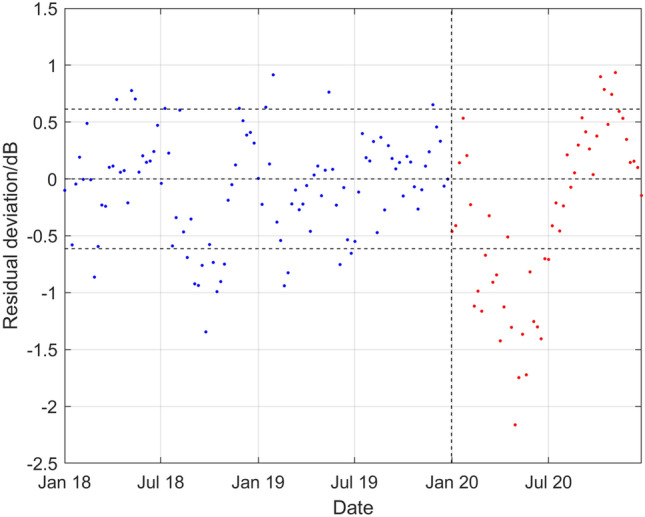
For the period January 2018 to December 2019 and during 2020, deviations of the observed weekly SPSDL data from the predicted values of the long-term and medium-term seasonal components of the GP that define the predictable part of the signal shown in Fig. 4.

Figure 4 shows that part of Fig. 2 restricted to the more recent period January 2018 to December 2019, the observed weekly SPSDL data $\left( {\tau_{j} ,\psi_{j} } \right),j = 1, \ldots ,n,$ collected in 2020 and the predicted values of SPSDL in 2020 provided by the GP model with their corresponding uncertainty band. The width of the uncertainty band is greater for predictions made at times further from the end of 2019 than for those made at times closer to 2019. Furthermore, the predictions in 2020 do not exhibit some of the short-term fluctuations present in earlier years. Nevertheless, the observed SPSDL data between January 2020 and July 2020 lie consistently close to the lower curve defining the uncertainty band for the predictions. Figure 5 shows, for the same time period as in Fig. 4, the deviations of the observed weekly SPSDL data from the predicted values of the long-term and medium-term seasonal components of the GP that define the predictable signal. To give some scale to the values, the two horizontal dashed lines are drawn at $\pm 2\hat{\sigma }_{0}$ to represent the estimated magnitude of the uncorrelated random effects. The short-term fluctuations that are missing from the signal are apparent in the deviations before 2020, but the drop in the observed SPSDL data between January 2020 and July 2020 compared to previous years is also clearly visible.

**Figure 6:**
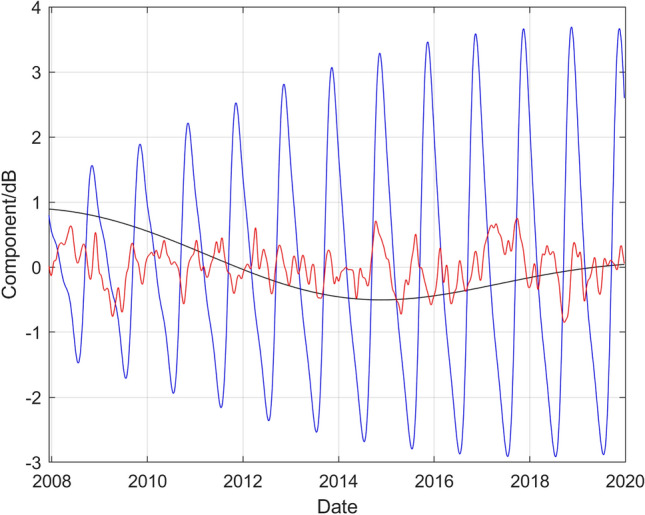
Comparison of the long-term, medium-term seasonal, and short-term components extracted from the results of the GP regression shown in Fig. 2. The black curve denotes the long-term component, the blue curve the medium-term seasonal component and the red curve the short-term component, respectively.

Figure 6 compares the long-term, medium-term seasonal, and short-term components extracted from the GP model. A notable feature is that the amplitude of the medium-term seasonal variation has increased substantially between 2008 and 2017 but thereafter the amplitude has remained essentially constant. Another feature is that the seasonal variation dominates the long-term and short-term components.

### Results for all stations

The analysis has been repeated for each combination of hydrophone (five in total) and frequency band (three in total), providing fifteen datasets. In each case, a decision about the adequacy of each fitted model is made on the basis of the corresponding standardised residual deviations. Although the extent to which the deviations closely follow a Gaussian distribution can vary between datasets, there is little evidence of (non-random) structure in those deviations. Occasionally, the estimate of a parameter can imply that a simpler model specification might suffice, for example, the estimate of $s_{M}$, a parameter describing the length-scale of changes in the seasonal fluctuations, can be large suggesting that variation in the periodic, seasonal behaviour is absent and does not need to be modelled. However, such model over-specification does not influence the predictions obtained, and it is not considered necessary to particularise the form of the model to each dataset.

**Figure 7:**
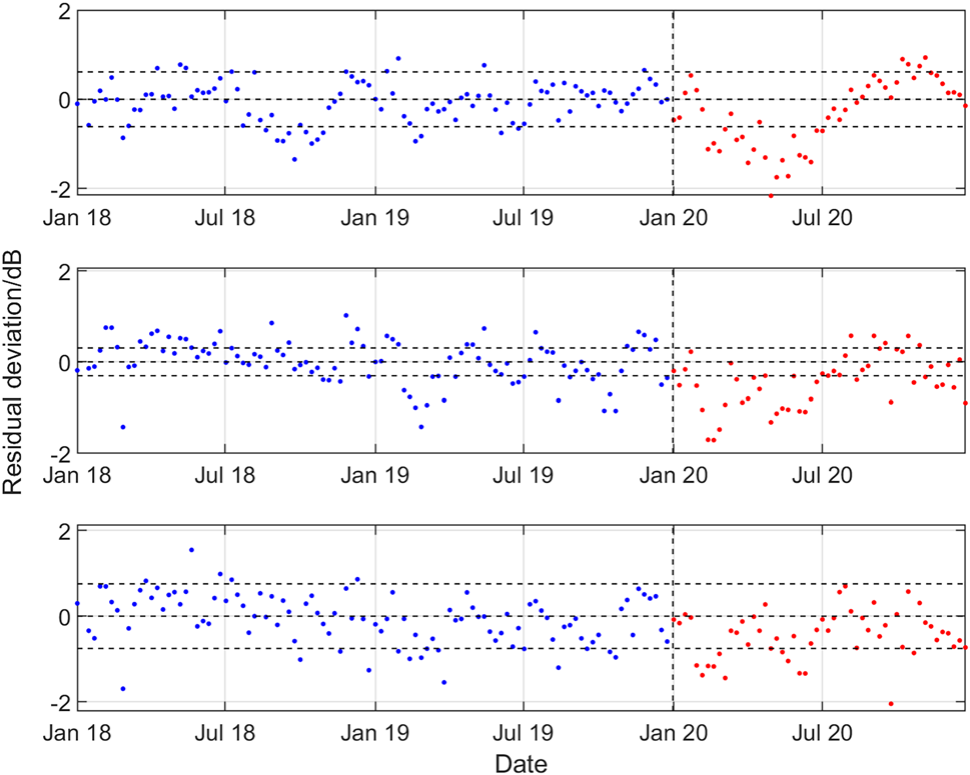
Results for the northern triplet hydrophone H11N1 data recorded at the CTBT Wake Island station, statistical level *P*_10_ and (top to bottom) low, medium, and high frequency bands. For the period January 2018 to December 2019 and during 2020, deviations of the observed weekly SPSDL data from the predictable part of the signal defined by the long-term and medium-term seasonal components of the GP model.

**Figure 8:**
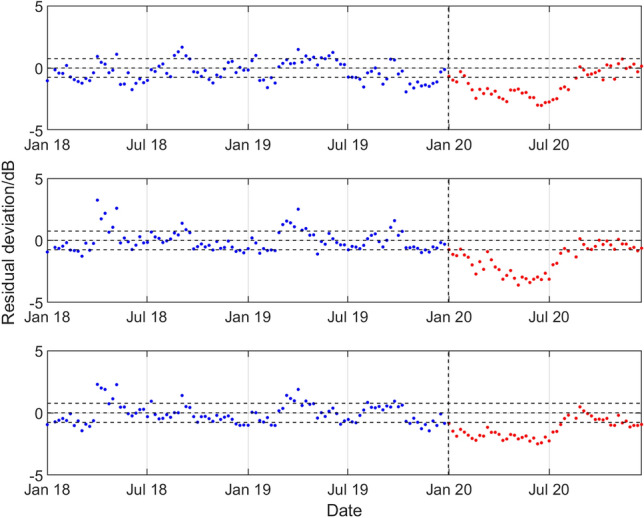
Results for the northern triplet hydrophone H10N1 data recorded at the CTBT Ascension Island station, statistical level *P*_10_ and (top to bottom) low, medium, and high frequency bands. For the period January 2018 to December 2019 and during 2020, deviations of the observed weekly SPSDL data from the predictable part of the signal defined by the long-term and medium-term seasonal components of the GP model.

**Figure 9:**
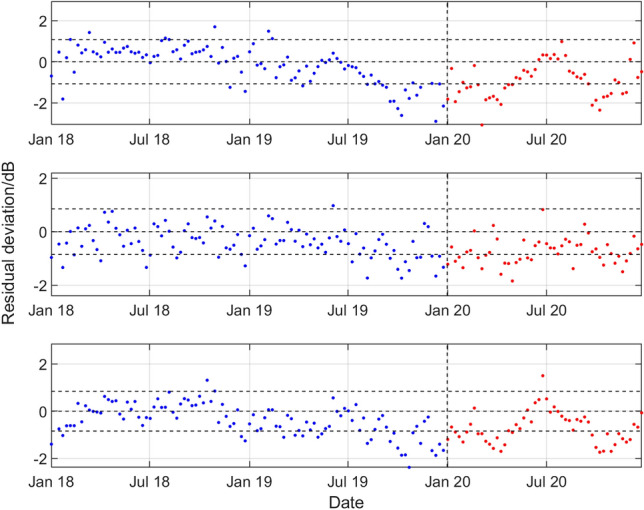
Results for the hydrophone H01W1 data at the CTBT Cape Leeuwin station, statistical level *P*_10_ and (top to bottom) low, medium, and high frequency bands. For the period January 2018 to December 2019 and during 2020, deviations of the observed weekly SPSDL data from the predictable part of the signal defined by the long-term and medium-term seasonal components of the GP model.

**Figure 10:**
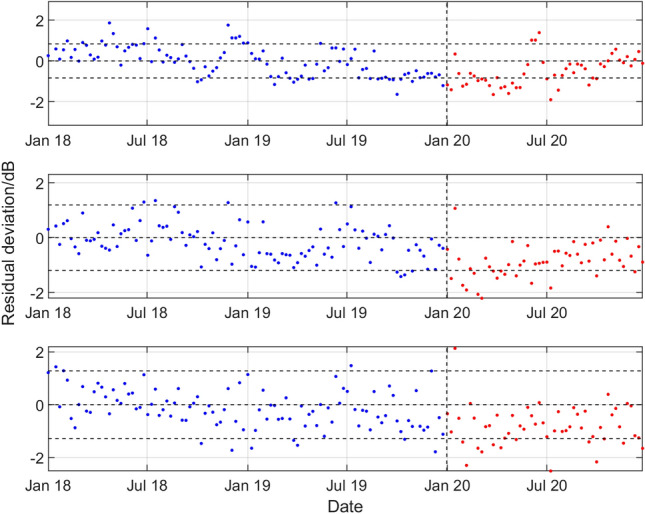
Results for the northern triplet hydrophone H03N1 data recorded at the CTBT Juan Fernandez Islands station, statistical level *P*_10_ and (top to bottom) low, medium, and high frequency bands. For the period January 2018 to December 2019 and during 2020, deviations of the observed weekly SPSDL data from the predictable part of the signal defined by the long-term and medium-term seasonal components of the GP model.

**Figure 11:**
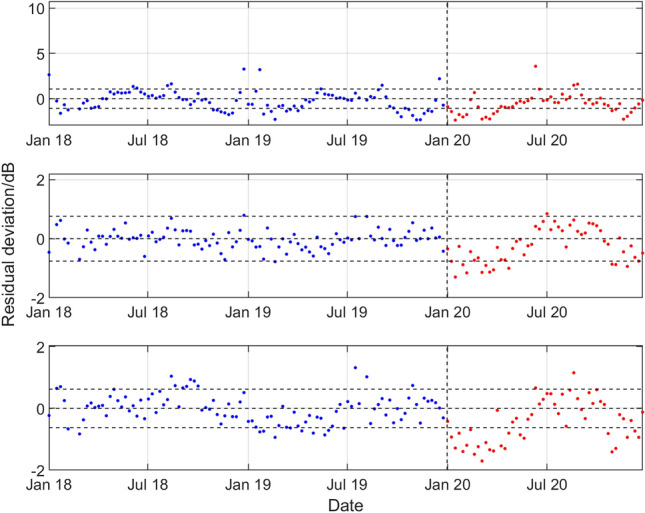
Results for the southern triplet hydrophone H08S1 data recorded at the CTBT Diego Garcia Island station, statistical level *P*_10_ and (top to bottom) low, medium, and high frequency bands. For the period January 2018 to December 2019 and during 2020, deviations of the observed weekly SPSDL data from the predictable part of the signal defined by the long-term and medium-term seasonal components of the GP model.

For hydrophone H11N1, Fig. 7 summarises the results for all three frequency bands, showing for the period January 2018 to December 2019 and during 2020 the deviations of the observed weekly SPSDL data from the predicted values of the long-term and medium-term seasonal components of the GP model that define the predictable signal, cf. Figure 5 that is reproduced in the top panel of Fig. 7. Figures 8, 9, 10, 11 then show similar results for, respectively, the number 1 hydrophones H10N1 (northern triplet), H01W1 (only triplet), H03N1 (northern triplet) and H08S1 (southern triplet) at the other four stations.

A reduction in the values of SPSDL in 2020 compared to previous years is very evident for H11N1 (although to a lesser extent for the high frequency band), for H10N1 in all frequency bands, and for H08S1 (although to a lesser extent for the low frequency band). In all these cases, the values of SPSDL appear to recover after June 2020 to the levels prior to 2020. For hydrophones H01W1 and H03N1 evidence for a reduction is less strong. However, there is some evidence, particularly for H01W1 and the low frequency band, that a reduction may have started before the end of 2019.

## Discussion

The results of the analysis are presented here for only one hydrophone from the triplet of hydrophones at each CTBT IMS hydroacoustic station. However, since the analysis of the data from the other two hydrophones at the stations (which are spaced only 2 km apart) shows similar results to those presented here, these have been omitted, and the particular choice of hydrophone treated in this study is not considered to have influenced the results.

The adequacy of the GP model fits to represent the data is in general satisfactory, with the diagnostics for the standardised residual deviations shown in Fig. 3 illustrating the quality of the representation of the data in that instance. The individual components of the model shown in Fig. 6 representing long-term, medium-term seasonal, and short-term behaviour illustrate the flexibility of the GP model. The long-term component, visible in Fig. 6, would not be represented well by a straight-line trend such as is used in some previous studies^34,38^. In addition, the amplitude of the medium-term seasonal variation has clearly evolved through the years, for example increasing in amplitude substantially between 2008 and 2017. The evolution of such features in the data is not easy to model with fixed functions such as polynomials or Fourier series.

Although the considerations above apply to the data for all three frequency bands and for all stations, the observed trends in the components of the model do vary between frequency bands, and between stations. For example, for the two higher frequency bands the annual seasonal fluctuations at Wake Island are more sinusoidal in nature, and do not show the same increase in the amplitude of the fluctuation with time. While the causes of the trends have not been established definitively, a strong correlation has been observed between the seasonal fluctuations and environmental factors such as sea surface temperature and, for Cape Leeuwin and Juan Fernandez Islands, sea ice and seasonal iceberg volume (generating low frequency noise during ice breaking and calving)^38^. For the dependency on sea surface temperature, a physical mechanism has been successfully postulated^33^ and it was shown that annual fluctuations in the sound level in the frequency range 63–125 Hz could be at least partially explained by seasonal variations in the sea surface temperature, which modulates the amount of sound energy trapped in the sound fixing and ranging (SOFAR) channel from sources on the sea surface (whether anthropogenic, such as ships, or natural such as breaking waves). The effect is due to the existence of a (seasonally dependent) critical grazing angle at the sea surface governing the ray paths are trapped by refraction and consequently are able to contribute to the global average sound field via the associated ocean acoustic waveguide. The effect depends upon whether the sound speed at the surface (at the location of the sources) is greater than that at the seabed, which depends on the ocean depth and the surface temperature^33^.

In addition to environmental factors, seasonality in the sound sources themselves can also influence the annual fluctuations. Examination of the narrowband spectra of the recorded data shows the calls of baleen whales in the frequency band 10–40 Hz for several of the stations with their presence being markedly seasonal in nature. In the case of Wake Island, it is this factor which gives rise to the increasingly non-sinusoidal fluctuations observed.

With the exception of the results for Cape Leeuwin (H01W1), the results of the analysis show a reduction in the sound levels for the first half of 2020, with something of a recovery in the second half of the year. A reduction is observed of between 0.7 and 2.0 dB for the lower two frequency bands at Wake Island between early February and the end of June. The reductions are particularly marked for Ascension Island (H10N1) for all three bands during the period early January to late July, with an average reduction of about 2 dB for all the bands, and up to 3 dB for the 40–70 Hz band in April and May. For Diego Garcia Island (H08), there are reductions in the first few months of 2020 of between 1.0 and 1.5 dB for the higher two frequency bands. Finally, for Juan Fernandez Islands (H03N1), the reductions are more modest, but evident in the middle frequency band.

The results shown here are in accord with the reports of localised level reductions in shallower coastal waters, where reductions of 1–2 dB have been observed and strongly associated with reduction in traffic^20,21,24^. There is no reason to expect similar reductions in shallow and deep-water studies because of the limited low frequency propagation in shallow water restricting the range over which the lowest frequencies produced by shipping can be detected, and it should be noted that coastal water studies monitoring the sound at frequencies up to the kilohertz range.

Ships are a significant anthropogenic source of ocean sound at frequencies below 100 Hz, and these reductions accord with the observed reductions in ship traffic in recent studies^10,11^. The studies surveyed calls at ports and all ships reporting their position and navigational status via AIS and showed an unprecedented drop in maritime traffic across all categories of commercial shipping. The greatest reductions in traffic were observed during the months of March to June 2020, with a gradual recovery later in the year. Reductions were reported in nearly 44% of the global oceans and in 77.5% of national waters during April alone, and greatest reductions were observed in container ships (13%) and passenger ships (42%). However, except for a few more detailed appraisals of local ship traffic, the data reported are cumulative global data sets. Although overall reduced traffic is evident for the surrounding ocean basins in which the CTBT hydroacoustic stations are located, there is not enough detailed traffic data available to demonstrate direct causation of the reduced sound levels through a deterministic model providing an estimate of the magnitude of the reduction.

Of course, ships are not the only anthropogenic source of sound at frequencies below 100 Hz, with geophysical surveying for oil and gas exploration being a significant source of impulsive sound^16^. Although these sources are intermittent, with surveys lasting several weeks at a time, their signals can be observed in the data from the CTBT stations when observed in spectrogram form. Published data is not available for the number of geophysical surveys in individual ocean regions, and so the type of analysis undertaken for ship traffic is not yet possible. However, a qualitative examination of the acoustic data for Ascension Island appears to show reduced activity from geophysical surveys in 2020, this station being able to detect such surveys occurring adjacent to the coasts of Africa and South America.

The exception to the observed reductions is seen at Cape Leeuwin, where a reduction is not easily detectable, and where the 2020 data in Fig. 9 could easily be considered as part of the same distribution of values around the predictable signal as for the pre-2020 data. The reason for this finding is not known, and detailed data for the shipping lanes off the west coast of Australia are not available to examine any correlation with marine traffic changes. However, a possible confounding factor to be considered for Cape Leeuwin is that southerly stations are known to have significant contributions from ice calving of sea ice and icebergs in the Antarctic^28,43–45^ which may be a more dominant influence than local shipping. In addition to the proximity of the station specific noise sources, another general factor influencing the sensitivity of the measured data to those sources may the potential for shadowing by the island which hosts the station (when the sources are on the opposite side of the island to the hydrophone station).

## Methods

There are different approaches to establishing a model $\hat{y}\left( t \right)$ for the purposes of estimating or predicting values $y\left( t \right)$ of SPSDL. One approach uses a model having a parametric form derived from an understanding of the physics underlying the deterministic and statistical processes that give rise to the data. However, for the problem considered here, such an approach is not considered practical because the physical processes are too complicated to be described in this way and because the quantity SPSDL is far removed, through a series of data reduction steps, from the quantity of acoustic pressure that is actually measured. An alternative to a physics-based model is to use an empirical model to describe SPSDL, and this was the approach previously applied to analyse the data collected at Cape Leeuwin^38^. That model included a straight-line function to describe a long-term trend, a function to capture medium-term seasonal variation that was assumed to be the same from year to year, and a fixed moving-average statistical function to describe the remaining short-term fluctuations that were expected to be correlated. Although the model was adequate for the earlier study^38^, it is not expected to be flexible enough to describe the data collected by the hydrophones at all CTBT stations. An inspection of the data for those stations^39^ suggests that the global trend is generally more complex than a simple straight-line function, and the seasonal variation and short-term fluctuations are expected to change from year to year in ways that are difficult to describe parametrically. Another possibility is to apply a purely data-driven approach in which a very flexible model, such as a Long-Short-Term-Memory (LSTM) neural network, is used. But such an approach, which ignores any available domain knowledge, can fail to generalise well to time periods where data is missing, and such generalisation is precisely what is needed here. Furthermore, the theory necessary to provide uncertainty information to accompany predictions on the basis of such data-driven models is not well-developed.

The approach taken in this study is a mixture of the parametric and data-driven (non-parametric) approaches described above. A stochastic model, which is not limited by a fixed functional form, in the form of a Gaussian Process (GP) is used but supplemented by domain knowledge both to inform the structure of the GP model as well as to provide prior knowledge about the parameters used in its specification. The approach is motivated by the application of GP regression to analyse data representing the concentration of atmospheric carbon dioxide at Mauna Loa^40^. This quantity exhibits similar characteristics in terms of long-term and seasonal trends to the quantity of SPSDL studied here, and the analysis is used as a template for the analysis described here. The use of GPs is reported in other underwater acoustics research studies^46,47^, but those applications, relating to sound-field reconstruction and source localization, are quite different from the application considered here.

A GP defines a probability distribution over functions parametrised by a variable such as time $t$ or spatial location. The definition of a GP denoted by $X\left( t \right)$ involves the specification of a mean function $\mu \left( t \right) = {\text{E}}\left[ {X\left( t \right)} \right]$ and a covariance kernel $k\left( {t,t^{\prime}} \right) = {\text{cov}}\left[ {X\left( t \right),X\left( {t^{\prime}} \right)} \right]$, where ${\text{E}}$ and ${\text{cov}}$ denote the expectation and covariance operators applied to discrete random variables. A GP can be interpreted in the following way: given a finite set of times $t_{i} ,i = 1, \ldots , m$, the finite set of random variables $X\left( {t_{i} } \right),i = 1, \ldots ,m$, are described by the multivariate Gaussian distribution ${\text{N}}\left( {{\varvec{\mu}},K} \right)$, where ${\varvec{\mu}}$ is a mean vector with elements $\mu \left( {t_{i} } \right)$ and $K$ is a covariance matrix with elements $k\left( {t_{i} ,t_{j} } \right)$. In particular, the covariance kernel controls the spread of possible function values at each time $t$ and how correlated are possible function values at different times $t$ and $t^{\prime}$, which in turn controls the shape of possible functions of the GP. It follows that the choice of covariance kernel is critical to ensuring that the GP is able to generalise to new data, which is needed to provide reliable and meaningful predictions.

The model used is

$$y_{i} = \hat{y}\left( {t_{i} } \right) + e_{i} = \overline{y} + \overline{y}\left( {t_{i} } \right) + e_{i} , i = 1, \ldots ,m,$$

where $\overline{y}$ is the average of the observed values $y_{i} ,i = 1, \ldots ,m$, $\overline{y}\left( t \right)$ is a zero-mean GP with covariance kernel $k\left( {t,t^{\prime}} \right)$, and $e_{i}$ is an independent random draw from a zero-mean Gaussian distribution ${\text{N}}\left( {0,\sigma_{0}^{2} } \right)$ with unknown variance $\sigma_{0}^{2}$ used to represent uncorrelated random effects. The covariance kernel is decomposed as

$$k\left( {t,t^{^{\prime}} } \right) = k_{L} \left( {t,t^{^{\prime}} } \right) + k_{M} \left( {t,t^{^{\prime}} } \right) + k_{S} \left( {t,t^{^{\prime}} } \right),$$

involving components representing characteristics of SPSDL at, respectively, long, medium (seasonal), and short timescales

The long-term trend is modelled using the squared-exponential covariance function:

$$k_{L} \left( {t,^{^{\prime}} t} \right) = \sigma_{L}^{2} \exp \left( { - \frac{{\left( {t - t^{^{\prime}} } \right)^{2} }}{{2s_{L}^{2} }}} \right).$$

The length-scale parameter $s_{L}$ in this function controls the timescale over which the trend is expected to vary appreciably. A large value of the parameter describes a trend that is close to a constant function, but more complex behaviour is also possible. The amplitude parameter $\sigma_{L}$ controls the vertical scale of variations in the trend.

The medium-term seasonal variation is modelled using the product of a squared-exponential covariance function and a periodic covariance function:

$$k_{M} \left( {t,t^{^{\prime}} } \right) = \sigma_{M}^{2} \exp \left( { - \frac{{\left( {t - t^{^{\prime}} } \right)^{2} }}{{2s_{M}^{2} }}} \right) \times \left( {\frac{{\kappa \left( {t - t^{^{\prime}} } \right) - \frac{1}{\pi }\mathop \smallint \nolimits_{0}^{\pi } \kappa \left( t \right)dt}}{{\kappa \left( 0 \right) - \frac{1}{\pi }\mathop \smallint \nolimits_{0}^{\pi } \kappa \left( t \right)dt}}} \right),$$

where

$$\kappa \left( {t - t^{^{\prime}} } \right) = \exp \left( { - \frac{2}{{s_{P}^{2} }}\sin^{2} \left[ {\frac{{\pi \left( {t - t^{^{\prime}} } \right)}}{p}} \right]} \right).$$

The inclusion of the squared-exponential term in this function allows the seasonal variation to change from year to year at a rate that is controlled by the length-scale parameter $s_{M}$. Furthermore, the period of the variation is controlled by the parameter $p$, which is fixed at *p* = 1 year, and the length-scale parameter $s_{P}$ controls the timescale of the variation within a period.

Finally, short-term fluctuations in the data, produced by sound sources lasting for timescales less than one year, are modelled using a rational quadratic covariance function:

$$k_{S} \left( {t,t^{^{\prime}} } \right) = \sigma_{S}^{2} \left( {1 + \frac{{\left( {t - t^{^{\prime}} } \right)^{2} }}{{2\alpha s_{S}^{2} }}} \right)^{ - \alpha } .$$

This function is used to represent a mixture of squared-exponential covariance functions having different length-scales, peaking at around $s_{S}$, with the index $\alpha$ determining the relative weighting between the different length-scales. (As $\alpha \to \infty$, the covariance function reduces to a squared-exponential covariance function with length-scale parameter $s_{S}$.)

It follows that the GP is determined by parameters ${\varvec{\theta}} = \left( {\sigma_{L} ,s_{L} ,\sigma_{M} ,s_{M} ,s_{P} ,\sigma_{S} ,s_{S} ,\alpha } \right)^{{\text{T}}}$ that, together with the standard deviation parameter $\sigma_{0}$, are unknown and are to be estimated from the available data $\left( {t_{i} ,y_{i} } \right),i = 1, \ldots ,m$. In addition to using domain knowledge to decide the form of the covariance kernel for the GP, prior knowledge about the parameters ${\varvec{\theta}}$, in the form of probability distributions $p\left( {\theta_{i} } \right)$ for the components $\theta_{i}$ of ${\varvec{\theta}}$, is included. Specifically, the parameters $s_{L} ,s_{M}$ and $s_{S}$, associated with the different components of the GP model, are assigned approximately rectangular distributions to ensure that the components capture correctly the long-term, medium-term seasonal, and short-term characteristics of SPSDL as intended. For example, it is expected that $s_{S} < 1$ year to represent short-term fluctuations at timescales of less than 1 year, whereas $s_{L} > 1$ year and $s_{M} > 1$ year, with $s_{M}$ measured, possibly, in decades if the periodic characteristics of SPSDL are stable with time. Furthermore, it is found that the inclusion of such prior knowledge is important as it helps to reduce confounding between the contributions from the different components of the covariance kernel.

Estimates of the parameters ${\varvec{\theta}}$ and $\sigma_{0}$ are determined by maximising $p\left( {s_{L} } \right)p\left( {s_{M} } \right)p\left( {s_{S} } \right)p({\varvec{\theta}},\sigma_{0} |{\varvec{y}})$ where ${\varvec{y}} = \left( {y_{1} , \ldots ,y_{m} } \right)^{{\text{T}}}$ and $p({\varvec{\theta}},\sigma_{0} |{\varvec{y}})$ is the marginal likelihood of the parameters conditional on the observed data, given by

$$\log p\left( {\theta ,\sigma_{0} {|}{\varvec{y}}} \right) = - \frac{1}{2}{\varvec{y}}^{T} \left( {K + \sigma_{0}^{2} I} \right)^{ - 1} {\varvec{y}} - \frac{1}{2}\log \left| {K + \sigma_{0}^{2} I} \right| - \frac{m}{2}{\text{log}}2\pi .$$

Here, $K = K\left( {\varvec{\theta}} \right)$ is the covariance matrix for the observed data with elements $k\left( {t_{i} ,t_{j} } \right)$ depending on ${\varvec{\theta}}$ and $I$ denotes an identity matrix. Using the estimates $\hat{\user2{\theta }}$ and $\hat{\sigma }_{0}$ so obtained as representative values, the value $\hat{y}\left( {\tau_{j} } \right)$ of SPSDL at time $\tau_{j}$ is described by the Gaussian distribution ${\text{N}}\left( {\hat{y}_{j} ,u_{j}^{2} } \right)$ with expectation

$$\hat{y}_{j} = \overline{y} + \hat{\user2{k}}_{j}^{{\text{T}}} \left( {\hat{K} + \hat{\sigma }_{0}^{2} I} \right)^{ - 1} {\varvec{y}},$$

constituting the required predicted level, and variance

$$u_{j}^{2} = \hat{k}\left( {\tau_{j} ,\tau_{j} } \right) + \hat{\sigma }_{0}^{2} - \hat{\user2{k}}_{j}^{{\text{T}}} \left( {\hat{K} + \hat{\sigma }_{0}^{2} I} \right)^{ - 1} \hat{\user2{k}}_{j} ,$$

giving the required standard uncertainty $u_{j}$ of the predicted level, where $\hat{K} = K\left( {\hat{\user2{\theta }}} \right)$ and $\hat{\user2{k}}_{j}$ is a vector containing the covariances $k\left( {\tau_{j} ,t_{i} } \right),i = 1, \ldots ,m$, evaluated in terms of $\hat{\user2{\theta }}$.

Although the preparation of the data includes the identification and removal of non-acoustic events, and the data for the statistical level $P_{10}$ is expected to be largely unaffected by short-term “loud” events, a final step in the analysis is to identify and remove outliers, considered to be values of SPSDL that are further than three times the standard uncertainty from the predicted level. These outliers generally correspond to isolated loud (anthropogenic or natural) events that are not expected to fit with the model. After removing such outliers, the steps of parameter estimation and model prediction described above are repeated. For the datasets studied here the number of such outliers never exceeds 1.1% of the size of a dataset.

It is noted that if, as is likely, the length-scale of the short-term component of the GP is small compared with the length of the time interval for extrapolation, which is one year, then that component will not contribute appreciably to the values of SPSDL predicted in that time interval although it will contribute to the uncertainty of those values. In that case the predicted values are determined by the long-term trend, which varies over a time period longer than one year, and the medium-term seasonal behaviour, which is constrained to be periodic with a period of one year and to change slowly over time periods longer than one year. In the application of GP regression to analyse data representing the concentration of atmospheric carbon dioxide at Mauna Loa^40^, the long-term and medium-term seasonal components are interpreted together as providing information about the “predictable signal”, whereas the short-term component and uncorrelated random effects are interpreted as providing information about the “unpredictable signal”. The same interpretation is made here and it influences the way that the results have been presented.

The GPML toolbox^48^ provides a MATLAB^®^ implementation of the methods for GP regression described in the text^40^, and the method of analysis used in this work has been applied using that toolbox.

## Data Availability

The data that support the findings of this study are available from CTBTO, but restrictions apply to the availability of these data, which were used under license for the current study, and so are not publicly available. Data are however available from the authors upon reasonable request and with permission of CTBTO. Enquiries may be directed to Georgios Haralabus, CTBTO.
